# Epicatechin Provides Antioxidant Protection to Bovine Spermatozoa Subjected to Induced Oxidative Stress

**DOI:** 10.3390/molecules24183226

**Published:** 2019-09-05

**Authors:** Eva Tvrda, Peter Straka, Drahomir Galbavy, Peter Ivanic

**Affiliations:** 1 Department of Animal Physiology, Faculty of Biotechnology and Food Sciences, Slovak University of Agriculture in Nitra, Tr. A. Hlinku 2, 949 76 Nitra, Slovakia; 2 Avelane Clinic, Krčméryho 2B, 949 01 Nitra, Slovakia; 3 Slovak Biological Services, Kremnička 2, 974 05 Banská Bystrica, Slovakia

**Keywords:** epicatechin, ferrous ascorbate, cryopreservation, antioxidants, bulls, spermatozoa, oxidative stress

## Abstract

Epicatechin (EPI) is a natural flavonoid with antibacterial, anti-inflammatory and anti-cancer properties. Furthermore, the molecule exhibits powerful reactive oxygen species (ROS) scavenging and metal-chelating properties. In this study, we assessed the efficiency of EPI to reverse ROS-mediated alterations to the motility, viability, DNA integrity and oxidative profile of bovine spermatozoa. For the first experiment, spermatozoa were washed out of fresh semen and exposed to 12.5 μmol/L EPI, 25 μmol/L EPI, 50 μmol/L EPI and 100 μmol/L EPI in the presence of ferrous ascorbate (FeAA) during a 6 h in vitro culture. For the second experiment, the ejaculates were split into aliquots and cryopreserved with a commercial semen extender supplemented with 12.5 μmol/L EPI, 25 μmol/L EPI, 50 μmol/L EPI, 100 μmol/L EPI or containing no supplement. Sperm motility was assessed using the computer-aided sperm analysis and the cell viability was studied with the metabolic activity test. ROS production was quantified using luminometry, and DNA fragmentation was evaluated using the chromatin dispersion test. Cell lysates were prepared at the end of the culture in order to assess the concentration of protein carbonyls and malondialdehyde. Exposure to FeAA led to a significantly reduced sperm motility (*p* < 0.001), mitochondrial activity (*p* < 0.001), but increased the generation of ROS (*p* < 0.001), as well as oxidative damage to proteins (*p* < 0.001), DNA (*p* < 0.001) and lipids (*p* < 0.001). EPI supplementation, particularly at a concentration range of 50–100 μmol/L, resulted in higher preservation of the spermatozoa vitality (*p* < 0.001). Furthermore, 50–100 μmol/L EPI were significantly effective in the prevention of oxidative damage to sperm proteins (*p* < 0.001), lipids (*p* < 0.001) and DNA (*p* < 0.01 in relation to 50 μmol/L EPI; *p* < 0.001 with respect to 100 μmol/L EPI). In the case of the cryopreserved spermatozoa, the administration of 50–100 μmol/L EPI resulted in higher sperm motility (*p* < 0.001) and mitochondrial activity (*p* < 0.001). ROS production, the number of protein carbonyls, lipid peroxidation as well as oxidative DNA damage were found to be significantly decreased particularly in samples cryopreserved in the presence of 100 μmol/L EPI (*p* < 0.001). Our results suggest that EPI could behave as an effective antioxidant which may prevent oxidative insults to spermatozoa, and thus, preserve their vitality and functionality. Nevertheless, its potential to achieve higher fertilization rates in reproductive technologies needs to be validated.

## 1. Introduction

The worldwide dissemination of ejaculates from a small number of stud males, particularly breeding bulls with superior genetic quality in order to inseminate a large number of females relies on meticulous semen processing protocols and suitable extenders [1]. Nevertheless, sperm preservation and storage come along with a continuous reduction of the vitality of male gametes, possibly due to the overgeneration of toxic reactive oxygen species (ROS) [2].

Certain levels of ROS produced by the mitochondrial respiratory chain are necessary for a normal sperm behavior, including capacitation, hyperactivation, acrosome reaction, oocyte fusion and fertilization [3]. However, a significant factor contributing to poor semen quality is oxidative stress (OS), which is a direct consequence of an imbalance between the ROS production and the antioxidant capacity of the biological system [4]. The concentration of polyunsaturated fatty acids (PUFAs) in sperm membranes is generally higher than in other cell types, which renders them to be highly vulnerable to oxidative damage, with a subsequent loss of membrane and DNA integrity, impaired motility and the onset of apoptosis [5]. Oxidative damage to the mitochondrial system, membrane and acrosome architecture may be a factor of major importance to explain the alterations in the motility and fertilization ability of spermatozoa subjected to high ROS concentrations [5,6]. When handled in vitro during assisted reproductive techniques, spermatozoa run the risk of being exposed to a supra-physiological level of ROS, which is a major parameter to be considered in order to evaluate the extent of OS [6,7].

One of the most suitable strategies for the prevention of ROS overproduction during semen processing is to enhance the antioxidant capacity of the extender, i.e., any liquid diluent added to semen in order to preserve its fertilizing ability. Though numerous studies have shown an enhancement of the sperm quality and fertilizing ability following the administration of traditional antioxidants, current attention has shifted to natural substances and extracts with a variety of beneficial and antioxidant properties. Indeed, there are various medicinal herbs known to manage diverse health issues and used as a part of traditional medicine [8,9].

Epicatechin ((–)-epicatechin; EPI) is a flavonoid compound belonging to the family of catechins, and primarily found in green tea, grapes, cocoa and chocolate [10,11]. Catechins are the most readily absorbable flavonoids [11] exhibiting a broad spectrum of anti-inflammatory, antibacterial, anti-cancer, anti-ischemic, and anti-thrombotic properties [11,12].

EPI has been described as a powerful scavenger of a wide spectrum of ROS, including superoxide and hydroxyl radicals, alongside being an effective LPO inhibitor [13]. Contradictory observations are, however, available with respect to the impact of EPI on male fertility. A number of in vivo [14,15], as well as in vitro studies [16,17] emphasize the ability of EPI to exhibit protective and antioxidant effects on male reproductive cells and tissues. Furthermore, EPI has been shown to reverse the reprotoxic effects caused by a variety of endogenous [15] or exogenous risk factors [18,19]. Other reports, however, indicate a negative role of EPI on the sperm function particularly in humans, suggesting a more rigorous assessment of the potentially toxic effects of EPI [20,21].

Earlier reports [22,23,24] have shown that ferrous ascorbate (FaAA) could act as a highly effective OS promoter for mammalian spermatozoa in the absence of the seminal plasma representing the primary antioxidant defense of ejaculates. Furthermore, to this date, no study has evaluated the effects of EPI on the vitality of frozen-thawed semen. Due to inconsistent evidence on the roles of catechins in male reproduction, there is an urge to explore their effects on spermatozoa further. This study was, therefore, designed to explore the in vitro impact of epicatechin on bovine spermatozoa exposed to oxidative stress induced by ferrous ascorbate. Subsequently, we focused on studying the effects of epicatechin on the activity and oxidative profile of cryopreserved bovine spermatozoa, in order to explore its potential to contribute to higher post-thaw semen quality.

## 2. Results

### 2.1. The Effect of Epicatechin on Spermatozoa Exposed to Ferrous Ascorbate

#### 2.1.1. Spermatozoa Motility

**Figure 1:**
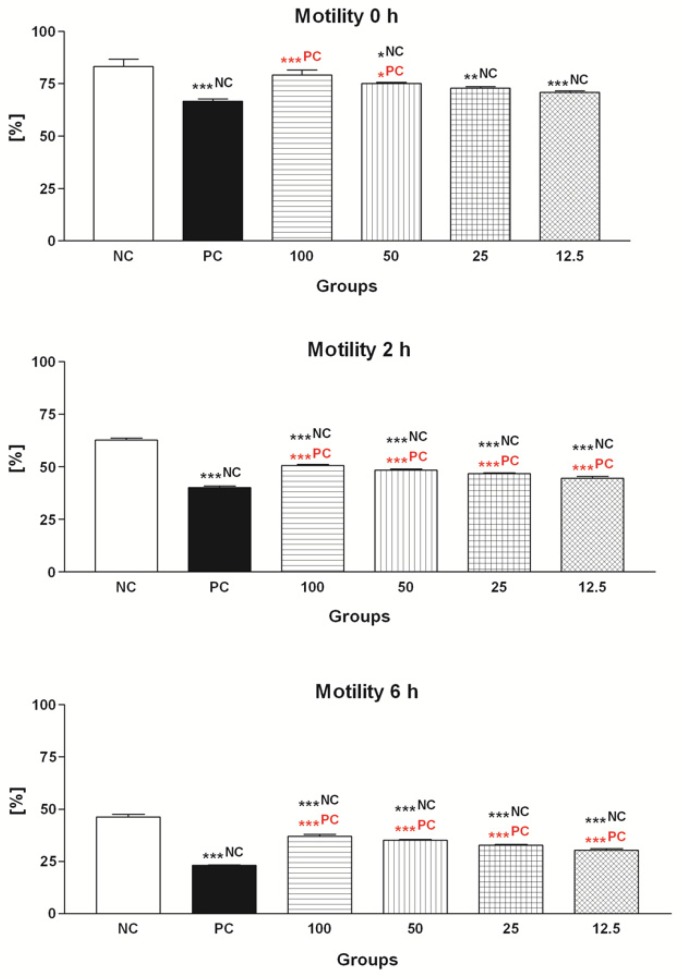
Motility of bovine spermatozoa exposed to four doses of epicatechin (EPI; μmol/L) in the presence of ferrous ascorbate (FeAA). The data were obtained from five independent experiments. * *p* < 0.05; ** *p* < 0.01; *** *p* < 0.001. NC—vs. negative control; PC—vs. positive control.

The data obtained from the computer-aided sperm analysis (CASA) revealed a significant (*p* < 0.001) decrease of the sperm motility over the course of the in vitro experiment as a result of FeAA administration (Figure 1). Exposure of spermatozoa pre-treated with FeAA to particularly 50 μmol/L EPI and 100 μmol/L EPI led to a significant improvement of the motion behavior (*p* < 0.001; 2 h and 6 h) when compared to the positive control (PC; FeAA control), although none of the chosen EPI concentrations were able to fully reverse the detrimental impact of FeAA on the sperm motility (*p* < 0.001 in comparison to the negative control—NC; 2 h and 6 h; Figure 1).

#### 2.1.2. Mitochondrial Activity

**Figure 2:**
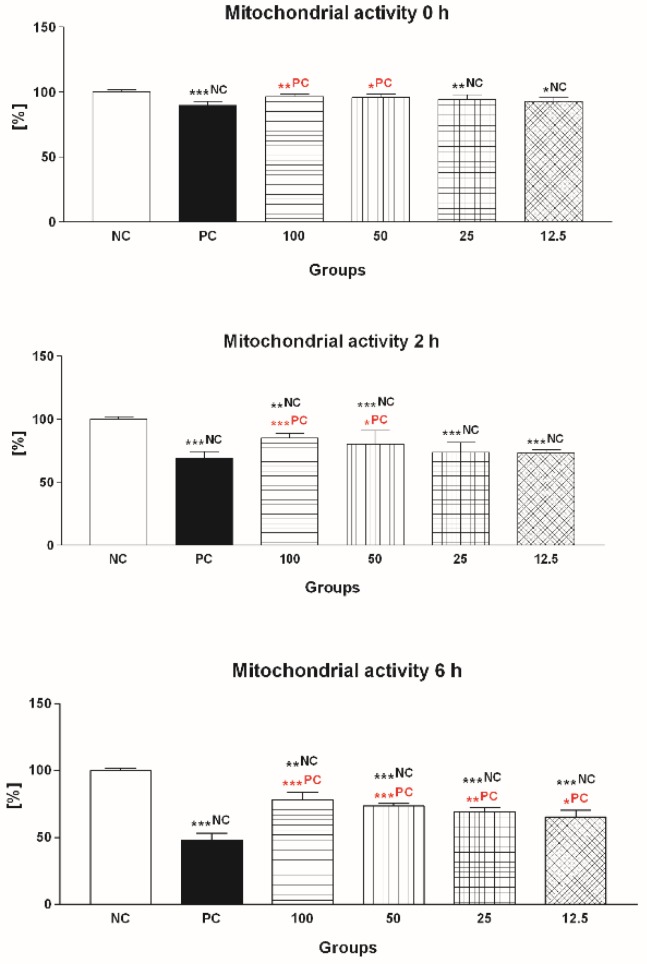
Mitochondrial activity of bovine spermatozoa exposed to four doses of epicatechin (EPI; μmol/L) in the presence of ferrous ascorbate (FeAA). Each bar represents mean (±SD) optical density as the percentage of the negative control (NC) set to 100%, and the data are expressed as a% of NC. The data were obtained from five independent experiments. * *p* < 0.05; ** *p* < 0.01; *** *p* < 0.001. NC—vs. negative control; PC—vs. positive control.

Correspondingly to the decreased motility, a significant decline of sperm mitochondrial activity was detected in PC during all timeframes of the experiment (*p* < 0.001; Figure 2). At the same time, 50 μmol/L EPI and 100 μmol/L EPI exhibited the ability to at least partially inhibit the mitochondrial dysfunction in the experimental groups subjected to FeAA treatment immediately after establishing the in vitro culture (*p* < 0.05 in the case of 50 μmol/L EPI; *p* < 0.01 with respect to 100 μmol/L EPI; 0 h), keeping their mitochondrial-protective effects to the end of the in vitro culture (*p* < 0.001; 6 h; Figure 2).

#### 2.1.3. Reactive Oxygen Species (ROS) Production

**Figure 3:**
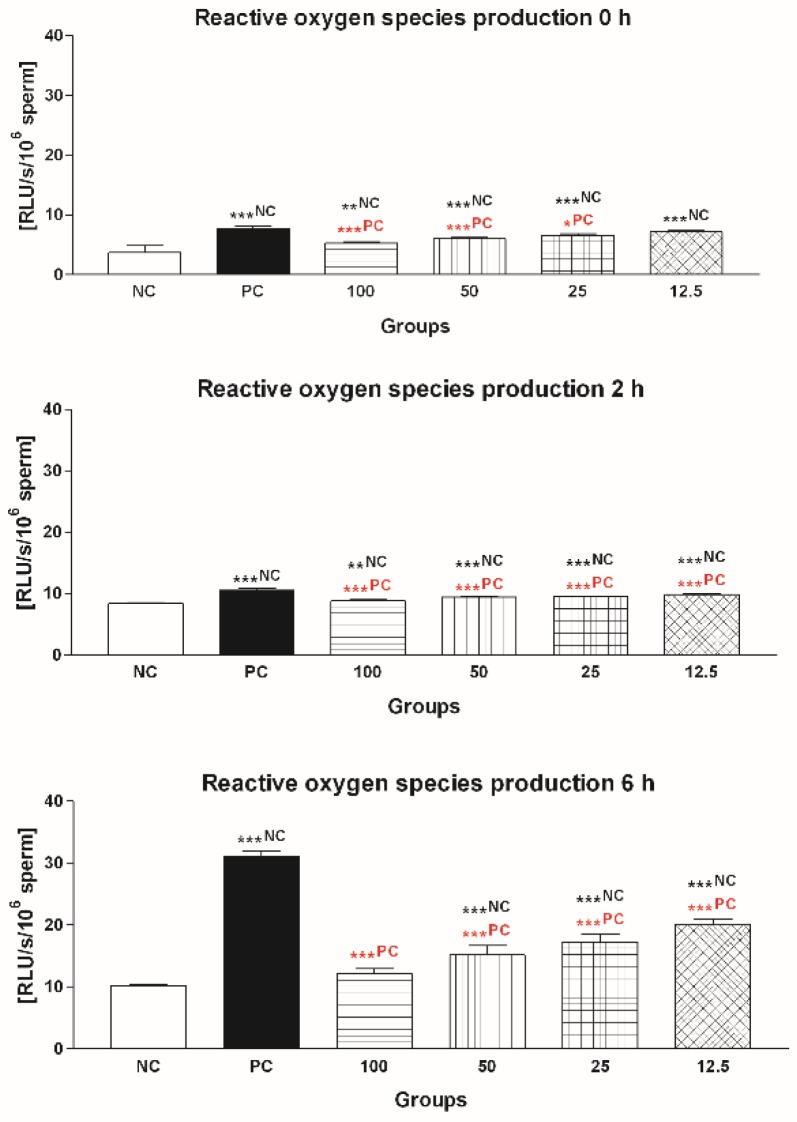
Reactive oxygen species (ROS) production by bovine spermatozoa exposed to four doses of epicatechin (EPI; μmol/L) in the presence of ferrous ascorbate (FeAA). The data were obtained from five independent experiments. * *p* < 0.05; ** *p* < 0.01; *** *p* < 0.001. NC—vs. negative control; PC—vs. positive control.

The decrease of the vitality of bovine spermatozoa exposed to FeAA was accompanied by a significant ROS overproduction (Figure 3). The amount of ROS became significantly increased immediately following FeAA supplementation in PC (*p* < 0.001, 0 h) and further rose at all assessment timeframes when compared to NC (*p* < 0.001, 2 h and 6 h). Free radical-quenching abilities of 100 μmol/L EPI were confirmed already at 2 h (*p* < 0.001). At 6 h it was noted that supplementation of EPI to the FeAA-pretreated culture did not lead to a complete reversal of the free radical overproduction; nevertheless, the flavonoid was capable to significantly decrease the concentration of ROS (*p* < 0.001) in comparison to PC (Figure 3).

#### 2.1.4. Sperm DNA Fragmentation

**Figure 4:**
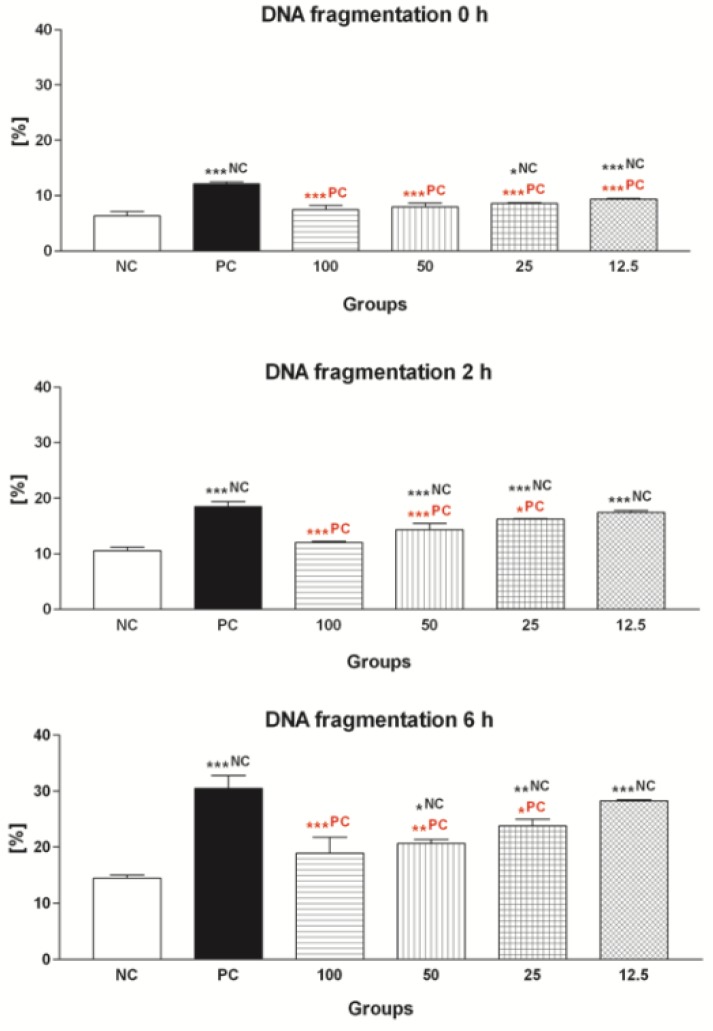
DNA fragmentation of bovine spermatozoa exposed to four doses of epicatechin (EPI; μmol/L) in the presence of ferrous ascorbate (FeAA). The data were obtained from five independent experiments. * *p* < 0.05; ** *p* < 0.01; *** *p* < 0.001. NC—vs. negative control; PC—vs. positive control.

As observed in Figure 4, FeAA had an instant detrimental effect on the DNA integrity of bovine spermatozoa, as the DNA fragmentation index was significantly increased in PC in comparison with NC (*p* < 0.001; 0 h). Damage to the sperm DNA became more pronounced with the increasing time of sperm incubation in the presence of FeAA (*p* < 0.001; 2 h and 6 h) when compared to the untreated group. The chromatin dispersion test revealed a significant protective effect of EPI against DNA fragmentation throughout the in vitro experiment. The polyphenol was able to prevent excessive DNA fragmentation of bovine spermatozoa exposed to FeAA particularly at doses of 25 μmol/L (*p* < 0.05; 2 h and 6 h), 50 μmol/L (*p* < 0.001 with respect to 2 h; *p* < 0.01 in the case of 6 h) and 100 μmol/L (*p* < 0.001; 2 h and 6 h).

#### 2.1.5. Oxidative Damage

**Figure 5:**
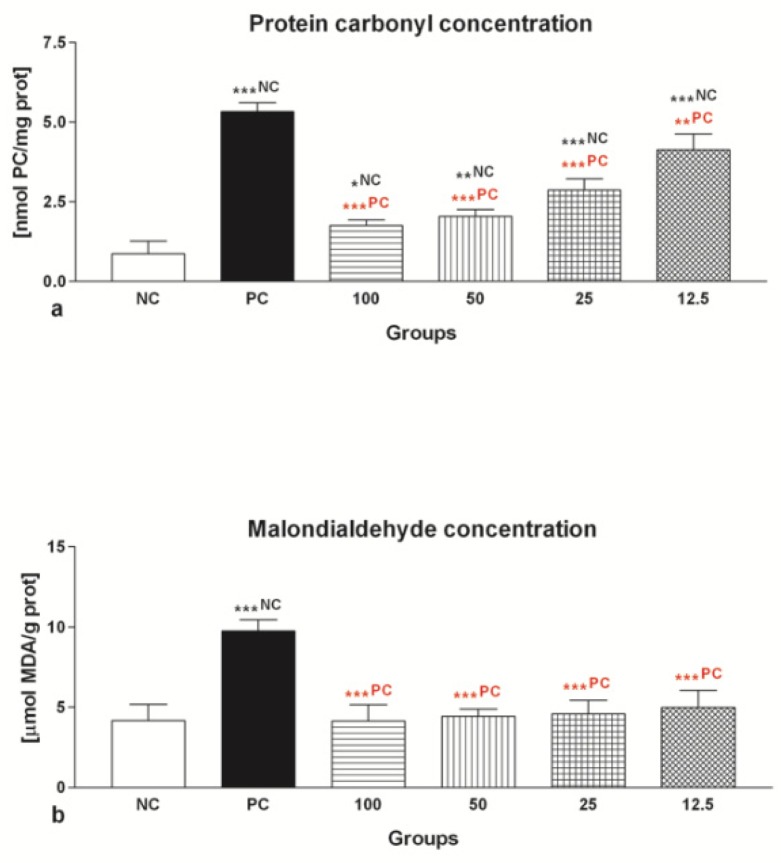
Oxidative damage to the proteins (**a**) and lipids (**b**) of bovine spermatozoa exposed to four doses of epicatechin (EPI; μmol/L) in the presence of ferrous ascorbate (FeAA). A—The data were obtained from five independent experiments. * *p* < 0.05; ** *p* < 0.01; *** *p* < 0.001. NC—vs. negative control; PC—vs. positive control.

The selected colorimetric analyses revealed a significant increase (*p* < 0.001) in the content of protein carbonyls (Figure 5a), as well as malondialdehyde (MDA; Figure 5b) in all groups exposed to FeAA in comparison to NC.

The protective effects of EPI on the protein and lipid biomolecules showed to be significant. The decrease of the number of protein carbonyls was particularly pronounced in the case of 25 μmol/L EPI, 50 μmol/L EPI and 100 μmol/L EPI (*p* < 0.001) in comparison with the PC (Figure 5a). Meanwhile, all selected EPI dosages were able to prevent excessive MDA formation when compared to the FeAA control (*p* < 0.001; Figure 5b).

### 2.2. The Effect of Epicatechin on Cryopreserved Spermatozoa

#### 2.2.1. Spermatozoa Motility

**Figure 6:**
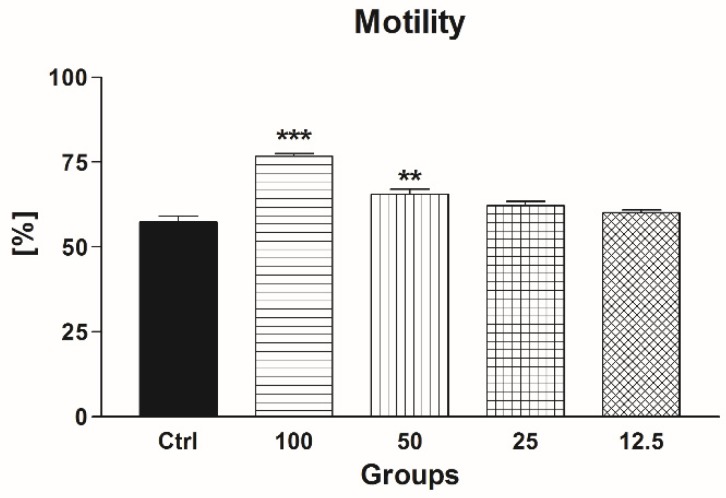
The effect of epicatechin (EPI; μmol/L) on the motility of frozen-thawed bovine spermatozoa. * *p* < 0.05; ** *p* < 0.01; *** *p* < 0.001.

Similarly to the data obtained from the previous motility analysis, we detected a significantly improved sperm motion activity in the case of groups supplemented with 50 μmol/L EPI and 100 μmol/L EPI (*p* < 0.001 in the case of 100 μmol/L EPI, *p* < 0.01 with respect to 50 μmol/L EPI) when compared to the control. No significant effects on the post-thaw motility were recorded in the groups exposed to 25 μmol/L and 12.5 μmol/L EPI in comparison with the control (Figure 6).

#### 2.2.2. Mitochondrial Activity

**Figure 7:**
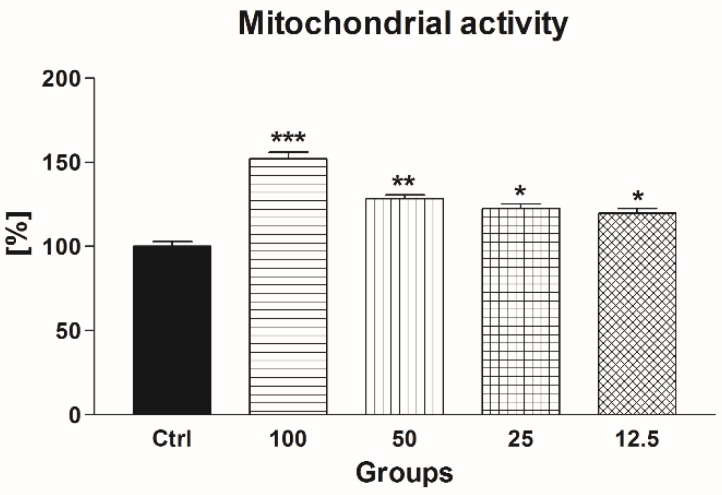
The effect of epicatechin (EPI; μmol/L) on the mitochondrial activity of frozen-thawed bovine spermatozoa. * *p* < 0.05; ** *p* < 0.01; *** *p* < 0.001.

Correspondingly to an improvement of post-thaw motility, a significantly increased mitochondrial activity was detected in all experimental groups exposed to EPI when compared to the untreated and cryopreserved control (*p* < 0.001 in the case of 100 μmol/L EPI; *p* < 0.01 in relation to 50 μmol/L EPI; *p* < 0.05 with respect to 25 μmol/L EPI and 12.5 100 μmol/L EPI; Figure 7).

#### 2.2.3. Reactive Oxygen Species (ROS) Production

**Figure 8:**
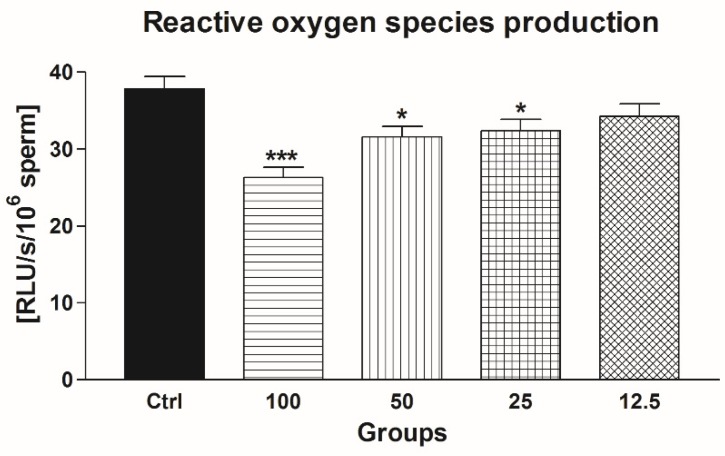
The effect of epicatechin (EPI; μmol/L) on the ROS production by frozen-thawed bovine spermatozoa. * *p* < 0.05; ** *p* < 0.01; *** *p* < 0.001.

Similarly to our previous observations, the ROS-quenching abilities of EPI were translated into a significantly lower ROS production in the groups supplemented with 25–100 μmol/L EPI (*p* < 0.001 with respect to 100 μmol/L EPI; *p* < 0.05 in the case of 25 μmol/L EPI and 50 μmol/L EPI) in comparison with the untreated control (Figure 8).

#### 2.2.4. Sperm DNA Fragmentation

**Figure 9:**
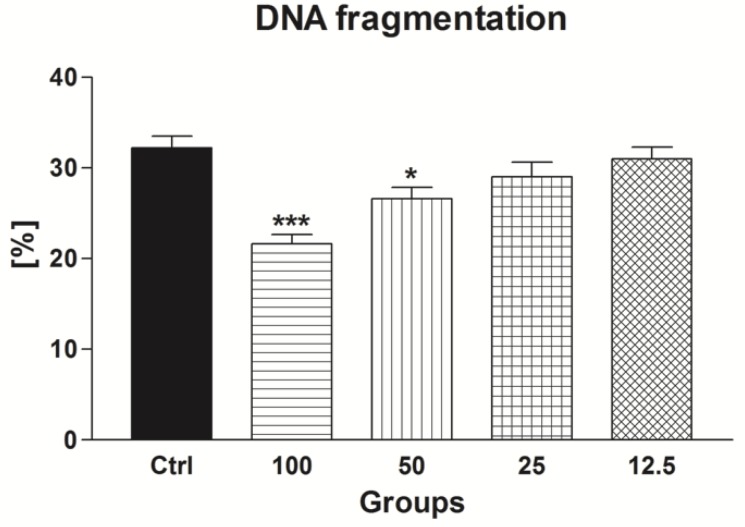
The effect of epicatechin (EPI; μmol/L) on the sperm DNA fragmentation by frozen-thawed bovine spermatozoa. * *p* < 0.05; ** *p* < 0.01; *** *p* < 0.001.

As revealed in Figure 9, EPI concentrations ranging between 50 μmol/L EPI and 100 μmol/L EPI were able to prevent significant damage to the DNA molecule to spermatozoa exposed to low temperatures (*p* < 0.001 with respect to 100 μmol/L EPI; *p* < 0.05 in the case of 50 μmol/L EPI). No significant changes were recorded in the case of 25 μmol/L EPI and 12.5 μmol/L EPI.

#### 2.2.5. Oxidative Damage

**Figure 10:**
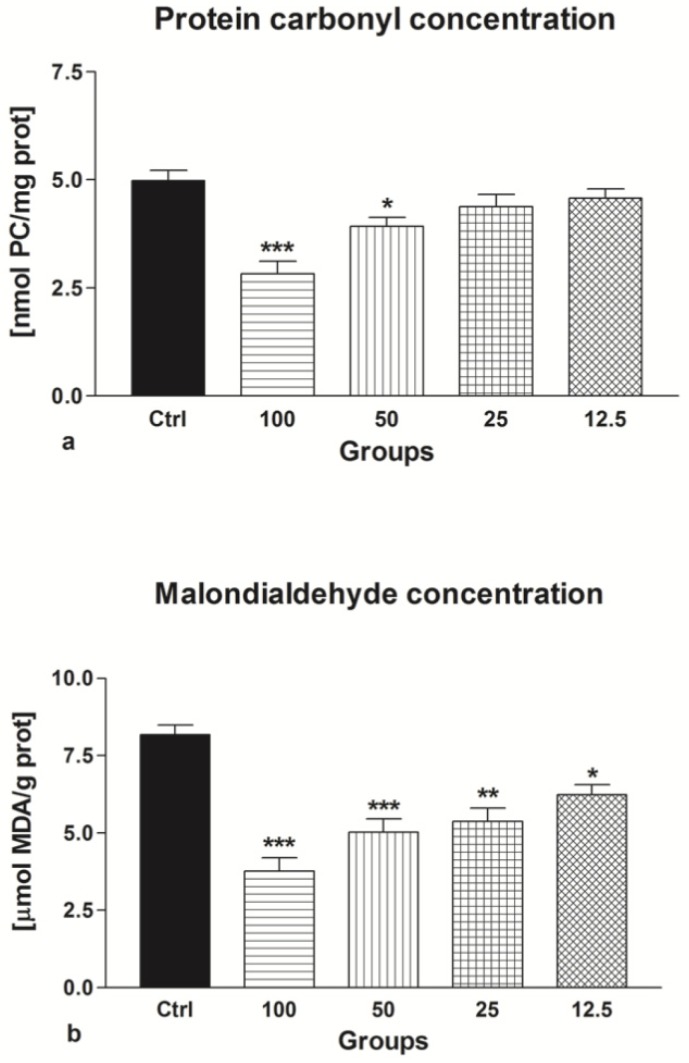
The effect of epicatechin (EPI; μmol/L) on the sperm protein oxidation (**a**) and lipid peroxidation (**b**) of frozen-thawed bovine spermatozoa. * *p* < 0.05; ** *p* < 0.01; *** *p* < 0.001.

Epicatechin exhibited cryprotective effects on the sperm proteins, as well as lipids. The decline of the number of protein carbonyls was significant in the case of 50 μmol/L EPI (*p* < 0.05) and 100 μmol/L EPI (*p* < 0.001) in comparison with the control (Figure 10a). Moreover, all selected EPI concentrations were able to cease excessive lipid peroxidation of frozen-thawed sperm when compared to the untreated control (*p* < 0.001; Figure 10b).

## 3. Discussion

Spermatozoa, unlike other cells, may be distinguished by a specific structure, function, and vulnerability to oxidative damage [5,25,26]. The first indications on an important role of ROS in the etiology of sperm dysfunction have surfaced from early reports associating the extent of lipid peroxidation (LPO) with a severe loss of sperm motion [5]. Similarly, the loss of motility has been observed on multiple occasions, when spermatozoa were subjected to a long-term incubation or cryopreservation, interrelating this phenomenon to the sperm ROS levels and LPO status at the end of the storage period [7]. As such, the use of ROS-promoters in andrology research has become a popular option to study in detail the specific physiological and pathological roles of ROS in male fertility.

While hydrogen peroxide is the most widely used substance to induce oxidative stress in sperm cultures in vitro [27,28], it has been shown that FeAA could act as an effective OS-promoting agent in mammalian spermatozoa [22,23,24]. The substance takes advantage on the specific characteristics of iron as a transition metal, thus, providing an appropriate environment for the Fenton and Haber-Weiss reaction [29] leading to oxidative degeneration of lipids, DNA and proteins [30]. Such deterioration of biomolecules essential to the sperm structural integrity may ultimately lead to motility depletion, mitochondrial dysfunction and cell death [1,5,13,20,23].

A significant disturbance of the sperm motion behavior and mitochondrial metabolism following exposure to FeAA and similar to our CASA report has been described in the case of mouse [22], deer [31] and equine [32] spermatozoa. Curiously, a study on stallion spermatozoa subjected to FeAA reported that high ROS amounts created as a result of FeAA administration led to a decreased sperm motility; however, no changes were detected in the case of the viability, acrosome integrity or mitochondrial activity [32].

It is well known that oxidative insults resulting from sperm freezing and thawing may result in a considerable decline in the motility, viability and membrane integrity of male gametes [5,7]. Cryopreservation of semen has been linked to axonemal aberrations, resulting in an abnormal motion behavior and mitochondrial function, ATP exhaustion, premature acrosome reaction and increased morphological alterations, due to oxidative insults to structural and functional biomolecules critical for the sperm survival [1,2].

An increasing body of evidence emphasizes on the fact that oxidative insults may have severe consequences on the nuclear and mitochondrial biomolecules, leading to the oxidative deterioration of DNA and proteins and accumulation of cytotoxic products, such as protein carbonyls or 8-oxo-2’-deoxyguanosine [31,33]. Lipids are the biomolecules most susceptible to oxidative damage, particularly in spermatozoa, which are known to contain exceptionally high amounts of PUFAs [5]. LPO may, in turn, cause alterations to the membrane architecture, followed by an increased incidence of lipid hydroperoxides, alkoxyl and peroxyl radicals, as well as toxic aldehydes, such as MDA [5,34]. The increased occurrence of markers of oxidative damage to the sperm proteins, lipids and DNA following FeAA supplementation observed in our study, as well as in previous reports [22,23,24,31] may ultimately lead to ATP depletion and inactivation of glycolytic enzymes [35,36] crucial for a proper sperm function.

Suppression of oxidative damage to the sperm structures through the administration of antioxidant substances with the ability to cease ROS generation or counteract oxygen toxicity, has been achieved with some success. Currently, numerous reports have suggested that natural antioxidants could improve the quality of stored semen [1,2,8,21,22,23,24]. The available evidence about the biological activity of epicatechin has provided the informative basis for our study, which was performed to assess the effect of selected EPI concentrations on the structural integrity, functional activity and oxidative profile of bovine spermatozoa exposed to FeAA or cryopreservation.

Catechin polyphenols, as the most important biologically active components of green tea, are known to be highly efficient ROS-scavengers and transition metal chelators. From a structural point of view, catechins, including EPI, contain hydrocarbon rings. The hydroxyl molecules are primarily responsible for the unique antioxidant behavior of catechins [37,38], which is 20 times higher than that of vitamin C. EPI has been described as a highly effective quencher of superoxide and hydroxyl radicals, offering antioxidant protection to biomolecules critical for the sperm function. Furthermore, the ability of catechins to chelate iron and copper has been reported to be similar to or even higher than other widely used chelators [39].

The improved sperm motility and mitochondrial activity, following EPI supplementation recorded in both experiments, disagree with Jamalan et al. [21]. According to their study, catechins (at 25–1000 μmol/L) did not exhibit any significant protective effect on human spermatozoa vitality following exposure to cadmiun, aluminum or lead. Moretti et al. [20] reported that while 200 μmol/L EPI and 400 μmol/L EPI had a compromising effect on human sperm motility and viability, exposure to lower concentrations of the polyphenol was associated with the maintenance of particularly progressive motility.

Inversely, previous observations by Greifova et al. [16] did suggest protective effects of EPI on bovine sperm motility when incubated over a period of 24 h. Similarly, Purdy et al. [40] state that various concentrations (25–100 μmol/L) of catechin led to a significantly improved of sperm motility of cooled caprine semen following a 96-h cultivation. A different study revealed that the motility and viability of boar spermatozoa supplemented with catechin (25 μmol/L and 50 μmol/L) was higher in comparison with the control group after 24 h and 48 h [41]. The contradictory findings on the behavior of EPI on the sperm motion may, therefore, depend on the studied species, as well as sperm processing technique. Furthermore, we must emphasize the fact that the dosage may be the single most important factor deciding whether catechins will exhibit beneficial or detrimental effects on sperm motion behavior.

Spermatozoa motility is closely associated with the mitochondria, which are helically arranged around the axoneme in the sperm mid-piece and play a key role in the energy production crucial for the sperm movement [42]. On the other hand, the mitochondrial system is the primary source of intracellular ROS, with the electron transport chain acting in an autoxidation process [20]. As such, any disturbance in the mitochondrial structure or metabolism may lead to severe dysfunction of the energy center of mitochondria with concomitant leakage of ROS into the extramitochondrial environment.

Supplementation of semen extenders with tea extracts rich in catechins has been shown to lead to a dose-dependent increase in the sperm viability [43], similarly to our assessment of the mitochondrial activity, superoxide and ROS production. Comparatively, lower concentrations of epigallocatechin gallate (2 μmol/L and 20 μmol/L) improved semen quality by increasing the viability and selected markers of human sperm capacitation through estrogen receptors [44]. Furthermore, Greifova et al. [16] indicate that exposure of bull spermatozoa to EPI concentrations lower than 100 μmol/L EPI led to an increased sperm mitochondrial function. Wittayarat et al. [45] state that vitamin C, along with 0.75 mg/mL green tea polyphenols, provided a significant stimulation of the motility and viability of stored canine semen.

A possible explanation for the beneficial effects of EPI on the mitochondrial metabolism may be provided by nuclear magnetic resonance spectroscopy studies suggesting a high affinity of catechins for the membranous structures of spermatozoa, particularly mitochondria [46]. Consequently, proper maintenance of the mitochondrial activity, with simultaneous prevention of ROS overproduction may result in a cell that is better suited to withstand a variety of internal or external assaults [47].

The antioxidant capacity of spermatozoa is closely related to male reproductive performance, as an appropriate antioxidant balance provides a suitable environment for the sperm function [26]. Beneficial effects of EPI supplementation on the stabilization of the sperm oxidative profile found in our study complement earlier reports focused on the antioxidant roles of catechins on the structure or function of the male reproductive system in animals and humans. Ding et al. [18] reported that catechin administration (50 mg/kg) to mice subjected to irradiation treatment protected against short-term germ cell loss and ameliorated the ionizing radiation-inflicted testicular OS. Furthermore, it was suggested that the polyphenol has a protective effect on the spermatogenic recovery and prevents germ cells from radiation-induced cell death, leading to a higher sperm concentration, motility and a decreased ROS generation [18].

Awoniyi et al. [14] state that the supplementation of Chinese green tea, rooibos and green tea supplements significantly improved the sperm concentration, motility and antioxidant capacity in rats treated with t-butyl hydroperoxide (TBHP), due to its ability to diminish ROS production and subsequent oxidative damage. The authors furthermore report that catechin administration resulted in an improvement of the antioxidant status together with stabilization of enzymatic antioxidants followed by a decrease of MDA generation in rat epididymal spermatozoa. Similarly to our findings, Awoniyi et al. [14] detected a significant increase in the antioxidant markers of the epididymal sperm in catechin treated rats. We may agree with the authors, that the presence of EPI may result in an enhanced endogenous detoxification capacity of spermatozoa, as GSH is known to interact with ROS, thus, reducing the risk of oxidative damage to biomolecules [48].

In this study, EPI significantly ceased oxidative damage to proteins, lipids and DNA as a possible result of its potential to interact with ROS before these can oxidize molecules critical for sperm survival. Similar protective effects were observed by Zanchi et al. [19] according to who green tea catechins significantly reduced LPO, protein carbonylation and DNA damage and restored GPx and glutathione S transferase activity in the testes of mice treated by cyclophosphamide. Catechins were also shown to be effective in the prevention of LPO and DNA damage in stored canine spermatozoa [45] and cryopreserved boar semen [49]. Also, data collected by Sapanidou et al. [50] showed that the incubation of thawed bovine spermatozoa in the presence of 2 μg/mL and 5 μg/mL of the green tea extract for 2 h led to a significantly better maintenance of viability and acrosome integrity, accompanied by a lower production of MDA.

Data from our assessment of the damage to the lipids are, however, in disagreement with Moretti et al. [20] who detected no significant effect of EPI on the extent of LPO of human spermatozoa subjected to TBHP. We may, therefore, speculate about the exact behavior of EPI on the sperm membrane, which is highly susceptible to LPO. EPI is an amphipathic molecule able to interact with proteins and phospholipids located in the plasma membranes. At the same time, its permeability across membranous structures and its lipid affinity depends on the degree of hydroxylation, molecular configuration, and the length of the side chain [51]. While EPI contains five hydroxyl groups, it behaved as a powerful antioxidant in some studies, and exhibited little to no antioxidant activity in others. It may be hypothesized that the stereochemistry of the hydroxyl group located on the C ring may have an impact on the antioxidant activity of EPI. Furthermore, unlike other catechins, EPI does not contain a carbonyl group, which may have a possible impact on the extent of the antioxidant properties of the molecule [38,52].

One issue that must be remembered, is the ever-increasing evidence on double-edged sword properties of EPI. While a number of studies have emphasized on the antioxidant properties of EPI, there are reports documenting its pro-oxidant activity, which may be caused by the metabolic conversion of catechins to pro-oxidant derivatives [53]. Due to their peculiar chemistry, these molecules are unstable and can contribute to ROS overgeneration by undergoing autooxidation. Sang et al. [54] speculate that the stability of catechins relies on their concentration, pH of the medium, the presence of oxygen, and the incubation temperature. All mentioned parameters must be, therefore, taken into consideration before EPI can be used as a supplement for sperm processing and preservation. Furthermore, the concentration range of EPI selected for our experiments may or may not be applicable to other species. Bovine spermatozoa used in our experiments, are known for higher resistance against stress conditions. In order to confirm the universal ability of EPI to prevent or reduce oxidative damage to spermatozoa, more in vitro studies focused on other mammalian species are highly necessary.

Lastly, in order to validate and translate our preliminary findings on the beneficial roles of EPI in the prevention of oxidative damage to bovine spermatozoa into its real implementation to the practice, it is highly recommended to compare its efficiency against other frequently used antioxidants, particularly in the process of bovine sperm cryopreservation. More importantly, it is imperative to evaluate the performance of EPI on the actual fertilization ability of bovine spermatozoa during artificial insemination or in vitro fertilization, either with fresh or with frozen-thawed semen.

## 4. Materials and Methods

### 4.1. Exposure to Ferrous Ascorbate

Forty ejaculates were collected from ten adult Holstein Friesian breeding bulls (Slovak Biological Services, Nitra, Slovak Republic) using an artificial vagina. Each sample included in the study had to meet the quality criteria given for the breed.

The experimental treatment was based on the protocol introduced by Bansal and Bilaspuri [24]. Each sample was centrifuged (800× *g*, 25 °C, 5 min), the seminal plasma was discarded, and the pellet was washed twice with 2.9% sodium citrate (SC; pH 7.4; Centralchem, Bratislava, Slovakia). The resulting spermatozoa were transferred into experimental media and diluted at 1:40 (for immediate assessments) or 1:20 (for cell lysis). Each sample was split into six equal fractions. One group (negative control; NC) carried 2.9% SC exclusively, while the second one (positive control; PC) contained ferrous ascorbate (FeAA) comprising 150 μmol/L FeSO_4_ (ferrous sulphate; FeSO_4_.7H_2_O; Sigma-Aldrich, St. Louis, MO, USA) and 750 μmol/L ascorbic acid (Centralchem), diluted in 2.9% SC. The remaining four groups were supplemented with 12.5 μmol/L, 25 μmol/L, 50 μmol/L or 100 μmol/L epicatechin (EPI; Sigma Aldrich) in the presence or absence of FeAA. The cell suspensions were incubated at 37 °C.

At culture times of 0 h, 2 h and 6 h, sperm motility and mitochondrial activity, ROS generation, intracellular superoxide production and DNA fragmentation were assessed in each group. At 6 h, each sample was centrifuged (800× *g*, 25 °C, 10 min), the media were removed, and the pellet was sonicated at 28 kHz for 30 s on ice using RIPA buffer (Sigma-Aldrich) containing protease inhibitor cocktail (Sigma-Aldrich). Subsequently, each sample was centrifuged (11,828× *g*, 4 °C, 15 min), and the residual cell debris was discarded. The resulting lysates were stored at −80 °C for the assessment of oxidative damage to the proteins and lipids, as well as the antioxidant profile.

### 4.2. Sperm Cryopreservation

For the second experiment, ejaculates from ten adult Holstein Friesian breeding bulls were used. Three samples were collected from each bull. Only samples with least 1 × 10^9^ sperm/mL and 70% progressive motility were included in the cryopreservation procedure.

Each sample was divided into five equal fractions and diluted to 11 × 10^6^ sperm/mL in a cryopreservation extender containing Triladyl (Minitube, Tiefenbach, Germany), 20% fresh egg yolk, Tris, citric acid, saccharose, glycerol, antibiotics, buffer solutions and diluted with distilled water. In the case of the experimental groups, the extender additionally contained 12.5 μmol/L EPI, 25 μmol/L EPI, 50 μmol/L EPI or 100 μmol/L EPI) previously dissolved in dimethyl sulfoxide (DMSO; Sigma-Aldrich), while the control group was supplemented with an equal amount of DMSO.

The extended ejaculates were loaded into 0.25 mL French straws, cooled down to 4 °C for 2 h and frozen at a programmed rate of −3 °C/min from 4 °C to −10 °C; −40 °C/min from −10 °C to −100 °C; −20 °C/min from −100 °C to −140 °C using the MT Freezer 2.0 for bull semen (Minitube). At last, the straws were submerged into liquid nitrogen (−196 °C) and stored for one month [55].

For the subsequent analyses, frozen straws were thawed in a 37 °C water bath for 20 s. All assays were performed on thawed spermatozoa following centrifugation (300× *g*, 25 °C, 5 min), washing and resuspension in phosphate-buffered saline (Dulbecco’s PBS; Sigma-Aldrich). In this experiment, we focused on the assessment of sperm motility, mitochondrial activity, ROS generation, as well as damage to the proteins, lipids and DNA. For the evaluation of the damage to proteins and lipids, cell lysates were prepared following the same procedure as described earlier.

### 4.3. Spermatozoa Motility Assessment

Sperm motility was assessed using the computer-aided sperm analysis (CASA) system (Version 14.0 TOX IVOS II.; Hamilton-Thorne Biosciences, MA, USA). Fluorescent illumination and the IDENT stain (Hamilton-Thorne Biosciences) were used for the sample processing. Each sample was placed into the Makler counting chamber (37 °C; Sefi Medical Instruments, Haifa, Israel) and at least 1000 cells were evaluated for motility (MOT; percentage of motile spermatozoa; motility > 5 μm/s; %) [55].

### 4.4. Sperm Mitochondrial Activity

The mitochondrial activity of spermatozoa was evaluated using the metabolic activity (MTT) test. Briefly, the MTT tetrazolium salt (3-(4,5-dimethylthiazol-2-yl)-2,5-diphenyltetrazolium bromide; Sigma-Aldrich) was dissolved in PBS (Dulbecco’s phosphate-buffered saline without calcium and magnesium; Sigma-Aldrich) at 5 mg/mL. Ten μL of the solution was added to the cell culture. Following incubation (2 h, 37 °C), 80 μL isopropanol (Centralchem) were added.

The optical density was determined at a wavelength of 570 nm against 620 nm as reference using a microplate spectrophotometer (Promega, Madison, WI, USA). The data are expressed as a percentage of the NC set to 100% [23].

### 4.5. Reactive Oxygen Species (ROS) Generation

The quantity of ROS in the samples was assessed by the chemiluminescence assay employing luminol (5-amino-2, 3-dihydro-1, 4-phthalazinedione; Sigma-Aldrich). The tested samples consisted of luminol (10 μL, 5 mM) and 400 μL of control or experimental sample. Negative controls were prepared by replacing the sperm suspension with 400 μL of each control or experimental medium. Positive controls included 400 μL of each medium and 50 μL of hydrogen peroxide (H_2_O_2_; 30%; 8.8 M; Sigma-Aldrich). Chemiluminescence was repeatedly measured on 48-well plates for 15 min using the Glomax Multi^+^ combined spectro-fluoro-luminometer (Promega). The results are expressed as relative light units (RLU)/s/10^6^ sperm.

### 4.6. Sperm DNA Fragmentation

Sperm DNA fragmentation was assessed using the Halomax commercial kit (Halotech DNA, Madrid, Spain). Tubes containing low-melting point agarose were placed in a water bath at 100 °C for 5 min to fuse the agarose, and subsequently transferred to an incubator at 37 °C. After 5 min of incubation, 20 μL of the sample was added to the agarose. Ten microliters of the mixture were pipetted onto slides pre-coated with agarose and covered with 20  ×  20 mm coverslips. The slides were then placed at 4 °C for 5 min to allow the agarose to turn into a microgel. The coverslips were gently removed, and the slides immersed horizontally into a lysis solution (5 min). Following washing in distilled water (5 min), the slides were dehydrated in 70% and 100% ethanol (2 min each) and air-dried [56].

All slides were stained using SYBR Green (2 μg/mL) (Sigma-Aldrich) in Vectashield (Vector Laboratories, Burlingame, USA) and a minimum of 300 spermatozoa per sample were scored using an epifluorescence microscope using a ×40 magnification objective (Leica Microsystems, Wetzlar, Germany). A minimum of 300 cells was scored in each slide. The extent of sperm DNA damage is expressed as %.

### 4.7. Oxidative Damage Assessment

The quantity of protein carbonyl was evaluated using the 2,4-dinitrophenylhydrazine (DNPH) method. One mL of the sample previously pretreated with trichloroacetic acid (TCA; 20% *w/v*; Sigma-Aldrich) was mixed with 1 mL DNPH (10 mM in 2 N HCl; Sigma-Aldrich) and incubated at 37 °C for 1 h. Following a second administration of 1 mL TCA, the suspension was incubated at 4°C for 10 min and centrifuged (11,828× *g*, 15 min). The resulting pellet was washed three times with 1 mL ethanol/ethyl acetate (1/1; *v/v*) and subsequently resuspended in 1 mL 6 M guanidine HCl (Sigma-Aldrich). The absorbance was measured at 360 nm, using 6 M guanidine HCl as a blank. The molar absorption coefficient of 22 000/M/cm was applied to calculate the number of protein carbonyls in each sample. Protein carbonyls are expressed in nmol/mg protein [57].

Lipid peroxidation (LPO) expressed through malondialdehyde (MDA) production was assessed using the TBARS assay, modified for a 96-well plate. Each sample was treated with 5% sodium dodecyl sulphate (Sigma-Aldrich) and subjected to 0.53% thiobarbituric acid (TBA; Sigma-Aldrich) dissolved in 20% acetic acid (pH 3.5; Centralchem) and subsequently boiled at 90–100 °C for 1 h. Afterwards, the samples were placed on ice for 10 min and centrifuged (1750× *g*, 10 min). The supernatant was used to measure the end product at 540 nm with the help of a microplate spectrophotometer (Promega). MDA concentration is expressed as μmol/g protein [55].

The protein concentration was determined using the DiaSys Total Protein (DiaSys, Holzheim, Germany) commercial kit and the RX Monza analyzer. The protocol follows the Biuret method, according to which copper sulphate reacts with proteins to form a violet blue color complex, and the intensity of the color is directly proportional to the protein concentration when measured at 540 nm.

### 4.8. Statistical Analysis

Statistical analysis was carried out using the GraphPad Prism program (version 5.02 for Windows; GraphPad Software, La Jolla, CA, USA). One-way ANOVA was used for statistical evaluations. Dunnett’s test was selected as a follow-up test to ANOVA, based on a comparison of every mean to a control mean, and creating a confidence interval for the difference between the two means. The comparative analysis for FeAA exposure was performed in the following sequence:

- Negative control (NC) was compared to the positive control (PC)
- Experimental groups were compared to both controls.

For the cryopreservation experiments, Dunnett’s test was used to compare the experimental groups to the untreated control. The level of significance was set at *** *p* < 0.001, ** *p* < 0.01 and * *p* < 0.05.

## 5. Conclusions

In conclusion, epicatechin exhibited the ability to prevent the decline of spermatozoa quality as a consequence of FeAA- or cryo-promoted oxidative damage. Epicatechin concentrations of 50 μmol/L and 100 μmol/L were particularly effective in protecting bovine male gametes against alterations caused by ROS overproduction through the prevention of oxidative degeneration, transformed into the preservation of sperm motility and metabolic activity. As such, epicatechin administration could become a suitable strategy to preserve the vitality of bovine spermatozoa and to diminish oxidative insults to their structural integrity and functional activity.
